# Ethnic differences in the burden of cardiovascular disease risk factors among adult residents of London: the TOGETHER study

**DOI:** 10.1186/s12916-026-04739-6

**Published:** 2026-03-03

**Authors:** Fotios Barkas, Malo Dirou, Kanika I. Dharmayat, Mahsa Mazidi, Antonio J. Vallejo-Vaz, Christophe A. T. Stevens, Amany Elshorbagy, Kausik K. Ray

**Affiliations:** 1 Imperial Centre for Cardiovascular Disease Prevention, Department of Primary Care and Public Health, School of Public Health, Imperial College London, School of Public Health Building, 90 Wood Lane, London, UK; 2 Department of Internal Medicine, Faculty of Medicine, School of Health Sciences, University of Ioannina, Ioannina, Greece; 3 Big Data and Analytical Unit, Imperial College, London, UK; 4 Department of Medicine, Faculty of Medicine, University of Seville, Seville, Spain; 5 Clinical Epidemiology and Vascular Risk, Instituto de Biomedicina de Sevilla, IBiS/Hospital Universitario Virgen del Rocío/Universidad de Sevilla/CSIC, Seville, Spain; 6 Centro de Investigación Biomédica en Red (CIBER) de Epidemiología y Salud Pública, Instituto de Salud Carlos III, Madrid, Spain; 7 Department of Physiology, Faculty of Medicine, University of Alexandria, Alexandria, Egypt

**Keywords:** Cardiovascular disease, Risk factors, Ethnicity, Race, Health check, Obesity, Diabetes, Dyslipidemia, Hypertension

## Abstract

**Background:**

Cardiovascular disease (CVD) remains the leading cause of premature death in England, with ethnic minority populations disproportionately affected, largely due to differences in socioeconomic factors, exposure and/or susceptibility to CVD risk factors. Midlife risk assessment does not fully account for observed variation in CVD incidence and mortality. Early and precise quantification of risk factor burden across diverse populations is therefore essential to inform targeted prevention strategies. This study assessed the prevalence of CVD risk factors in apparently healthy individuals residing in London.

**Methods:**

This cross-sectional study included CVD-free individuals aged 30–90 years residing in London and registered with general practices using the Egton Medical Information Systems (EMIS) electronic health record system. Unadjusted, crude estimates of traditional CVD risk factors were assessed across participants of different ethnicities who underwent a CVD risk assessment between 2009–2020.

**Results:**

Among 607,327 registered individuals, 83,414 were included (52.0% women, median age 45 [IQR:36–48] years). Ethnic distribution was as follows: White (43.6%), Asian (30.1%), Black (9.7%), Chinese/Other (4.0%), Mixed (2.1%). Overall, 7.8% were current smokers, 31.5% had obesity (universally defined as body mass index (BMI) ≥ 30.0 kg/m^2^), 48.5% had elevated blood pressure (BP ≥ 140/90 mmHg), 44.9% had hypercholesterolemia (≥ 5.0 mmol/l), 28.2% had elevated triglycerides (TG) ≥ 1.7 mmol/l, and 25.9% had low high-density lipoprotein cholesterol (HDL-C < 1.0/1.3 mmol/l for males/females, respectively). Smoking prevalence was highest among White individuals (9.7%). Obesity prevalence varied across groups, with higher proportions in Black participants (42.3%) and lower in Asian individuals (26.1%). Elevated BP was recorded more frequently in Mixed (54.9%) and Black (53.0%) participants and less frequently in those classified as Chinese/Other (42.7%). Total cholesterol ≥ 5.0 mmol/L was more commonly documented in Mixed (56.8%) and White (49.8%) participants. Higher proportions of Asian individuals had elevated TG (30.9%) and low HDL-C (31.6%), while corresponding proportions were lower among Black participants (14.4% and 19.5%, respectively).

**Conclusions:**

This large-scale analysis of a diverse population suggests variation in CVD risk factor burden among relatively young individuals without CVD. While not implying causality, these findings reflect inequalities between ethnic groups and support an appraisal of early, tailored, and equitable public health policies to improve CVD risk management across diverse populations.

**Supplementary Information:**

The online version contains supplementary material available at 10.1186/s12916-026-04739-6.

## Background

Despite advancements in prevention and control of underlying risk factors, cardiovascular disease (CVD) remains the major cause of disability and mortality worldwide, including the United Kingdom (UK) [1]. Based on the most recent British Heart Foundation report, CVD accounted for 174,693 annual deaths in the UK (26% of all deaths recorded), contributing to rising healthcare costs, estimated at up to £12 billion each year [2]. Although most CVD-related deaths occur in older adults, premature CVD-related mortality is a significant concern in the UK, with over 48,697 individuals dying annually from CVD causes before the age of 75 [3] and disproportionally affecting people of South Asian and Black ethnicity [4, 5].

Much of the existing UK evidence describing ethnic differences in risk of CVD is derived from studies conducted decades ago or from cohorts with limited ethnic diversity, restricted geographic coverage, or selected populations [6–16]. These studies had attributed observed disparities in cardiovascular outcomes largely to differences in exposure to and/or susceptibility to traditional CVD risk factors [5], as well as to variations in socioeconomic deprivation, health literacy and access to healthcare services [17]. However, the UK population has become substantially more ethnically diverse in recent decades, particularly in urban centres such as London, and patterns of migration, healthcare delivery, and risk factor detection have evolved with national prevention plans delivered mostly through primary care. As a result, the contemporary relevance and generalizability of earlier findings to today’s ethnically diverse general population in urban centres where the bulk of the population resides is increasingly uncertain.

National CVD risk assessment and management initiatives, including the Health Check programme by the National Health Service (NHS) in the UK, invite apparently healthy middle-aged individuals for evaluation of major CVD risk factors, with the aim of preventing coronary heart disease (CHD), stroke, diabetes, and kidney disease, as well as addressing ethnic disparities in CVD prevention [18]. However, such programmes have faced criticism due to low uptake in the general population and ongoing debate about the effectiveness of population-based health checks, with some arguing that opportunistic approaches may be more beneficial [19–23]. That said, traditional modifiable CVD risk factors measured in midlife do not fully account for variations in the risk of CVD observed across ethnic groups, suggesting that gaps in detection, management, or cumulative risk exposure from risk factors appearing earlier in life may undermine the aspirational equity of “one-size” population-level CVD prevention strategies [5, 8, 24].

The objective of the TOGETHER study (Imperial and The London General Practice-based Investigation of Cardiovascular Health and Risk Factors among Diverse Populations) was therefore to comprehensively assess the prevalence of CVD risk factors among ethnically diverse individuals free from CVD aged 30–90 years residing in London. By focusing on a highly diverse urban population and utilizing recent data, this study aims to generate contemporary evidence to potentially help inform gaps in public health strategies aimed at reducing ethnic disparities in cardiovascular health in the UK, in the setting where most preventive strategies are initiated.

## Methods

### Study design, setting, and participants

Protocol design has been previously reported [25]. This was a cross-sectional study leveraging routinely collected primary care electronic health records (EHR) in GP across London that used the Egton Medical Information Systems (EMIS) Web or EMIS LV platforms and agreed to participate in the present study. The observation period was from 1 January 2009 to 31 December 2020.

Eligible participants were adults aged 30–90 years and had an NHS Health Check or an opportunistic cardiovascular risk assessment by the participating GP during the study period. While the NHS Health Check programme is offered to apparently healthy adults aged 40–74 years without established CVD [18], we deliberately expanded the age range to 30–90 years to explore CVD risk factors across the wider life-course and to minimise potential biases related to the uptake or recording of NHS Health Checks in different groups.

Individuals were excluded if they were < 30 or > 90 years at the index visit; had established CVD documented at or before the index visit (defined as a recorded diagnosis of coronary heart disease, cerebrovascular disease, or peripheral artery disease); lacked an eligible GP visit with a recorded cardiovascular risk assessment or had insufficient clinical/risk-factor data in the EHR.

### Data sources

This study utilized routinely collected, structured primary care data from GPs in London recorded using the EMIS Web or EMIS LV EHR systems [25]. EMIS is one of the major providers of EHR software to GP practices in the UK, supporting standardised recording of clinical, prescribing, and administrative information. Anonymised data from the participating GP was extracted centrally by EMIS and securely transferred to encrypted servers maintained by the Big Data Analytics Unit (BDAU) at Imperial College London. Prior to analysis, data underwent cleaning and validation, including random record checks and targeted queries addressed to EMIS and participating GP practices to enhance data accuracy and completeness.

The extracted dataset included the following variables: demographics (age, sex, self-reported ethnicity, and postcode); smoking status; reported diagnoses of arterial hypertension, diabetes, obesity, dyslipidemia by the physicians; as well as anthropometric measurements (height, weight, body mass index (BMI)), blood pressure (BP) readings and lipid measurements.

Information on risk factors and diagnoses was captured using clinical codes extracted from EMIS, enabling standardised identification across practices. These clinical coding systems, routinely used in UK healthcare to record symptoms, diagnoses, and procedures, were used to define the inclusion and exclusion criteria, as well as the outcomes of interest (Additional File 1: Fig. S1).

### Outcome measures

The outcome measures were estimates of CVD risk factors captured from the index (first recorded) visit during the study period. These included self-reported smoking status, physician-reported diagnosis of obesity, diabetes, hypertension, and dyslipidaemia, as well as measured BMI, BP, and recorded laboratory measurements of total cholesterol, TG, high-density lipoprotein cholesterol (HDL-C), low-density lipoprotein cholesterol (LDL-C), and non-high-density lipoprotein cholesterol (non-HDL-C).

BMI categories were defined based on World Health Organization (WHO) cut-offs: obesity (BMI ≥ 30 kg/m^2^), overweight (BMI 25–29.9 kg/m^2^), healthy weight (BMI 18.5–24.9 kg/m^2^), and underweight (BMI < 18.5 kg/m^2^) [26]. Without accounting for participants’ concomitant therapies, measured BP values were grouped into the following categories: normal BP (systolic BP (SBP) < 130 mmHg and diastolic BP (DBP) < 85 mmHg); high-normal (SBP, 130–139 mmHg or DBP, 85–89 mmHg); and elevated (SBP ≥ 140 mmHg or DBP ≥ 90 mmHg) [27]. The cut-off for elevated total cholesterol (≥ 5.0 mmol/L) was defined according to WHO recommendations, while a LDL-C cut-off of ≥ 4.9 mmol/L was used, as values at or above this threshold are more likely to indicate familial hypercholesterolemia [28]. Triglycerides (TG) over 1.7 mmol/L were considered elevated, whereas HDL-C values below 1.0 mmol/L for males or < 1.3 mmol/L for females were considered low [28].

The outcomes of interest were examined across ethnic groups. Ethnicity was self-reported and recorded in EMIS using SNOMED/Read codes, which were then mapped to the following aggregated categories according to the 2001 UK Census: White (White British, White Irish, Other White); Asian or Asian British (Indian, Pakistani, Bangladeshi, Other Asian); Black or Black British (Black Caribbean, African, Other Black); Mixed (White and Black Caribbean, White and Black African, White and Asian, Other Mixed); and Chinese or Other Ethnic Group (Arab or any other ethnic group) [29]. The “not stated” category reflects individuals without a recorded ethnicity code. In addition, stratification was performed by age group (by 10-year intervals) and areas of residence, classified according to self-reported postcodes by the participants. Residential areas with ≥ 2000 participants were analysed separately, comprising Bromley (BR), Harrow (HA), Northwest London (NW), Southeast London (SE), Southwest London (SW), and Southall (UB). All remaining postcodes with fewer than 2000 participants were aggregated and reported as “other areas” (Additional File 1: Fig. S2).

### Ethics approval

The TOGETHER study protocol received ethical approval (Ref: 17-ES-0104) from the Joint Research Compliance Office and the Imperial College Research Ethics Committee (Imperial College London, UK). Participation by general practices required Research and Development approval from the respective NHS Trusts. The study adhered to the ethical principles of the Declaration of Helsinki [30].

### Statistical analyses

Continuous variables are presented as medians with interquartile ranges (IQR) (25th and 75th percentiles); categorical variables are summarized as frequencies and percentages. Group comparisons were performed using the Wilcoxon rank-sum test for continuous variables and Pearson’s χ^2^ test or Fisher’s exact test, as appropriate for categorical variables. Because multiple variables were evaluated across ethnicity categories, a Bonferroni correction was applied to account for the number of tests performed. Continuous measurements and their corresponding categorical definitions were treated as representations of the same underlying variable and were therefore counted once, yielding a total of 19 tests. The variables assessed were as follows: sex; age at registry entry; BMI (kg/m^2^)/BMI categories; systolic BP (mmHg)/blood pressure categories; diastolic BP (mmHg)/blood pressure categories; total cholesterol (mmol/L)/total cholesterol ≥ 5.0 mmol/L; TG (mmol/L)/TG ≥ 1.7 mmol/L; HDL-C (mmol/L)/HDL-C below sex-specific thresholds; LDL-C (mmol/L)/LDL-C ≥ 4.9 mmol/L; non-HDL cholesterol (mmol/L); smoking status; arterial hypertension; diabetes; obesity; dyslipidemia; and use of lipid-lowering, glucose-lowering, or blood pressure-lowering medications. The adjusted significance threshold was set at *α*′ = 0.05/19≈0.0026, and results were interpreted using this corrected level. Uncorrected *p*-values are presented in the tables and figures, with statistical significance determined according to the Bonferroni-adjusted threshold. All analyses report unadjusted, crude estimates of the outcomes of interest across ethnic groups. No missing data imputation was performed. For each outcome of interest, we performed variable-specific complete-case analyses, including all participants with a recorded value for that variable. All analyses were conducted using R version 4.2.1 (Windows).

## Results

Of the 607,327 individuals registered with participating GP practices, 83,414 eligible participants were included in the present study, of whom 1595 had a coded complete NHS Health Check (Additional File 1: Fig. S1).

### Demographic characteristics

Demographic characteristics are summarized in Table 1. The largest ethnic group was White (36,331; 43.6%), followed by Asian (25,148; 30.1%); Black (8054; 9.7%); Chinese/Other (3347; 4.0%); and Mixed ethnicities (1793; 2.1%) (Additional File 1: Fig. S3). Overall, median age was 45 (IQR 36–48) years, with 62% of participants aged below 50 years, and 52% were women. Most participants resided in Harrow (45,688; 55%) and NW London (17,289; 21%).

**Table 1:** Demographic characteristics of total population and study participants by ethnicity

	**Total subjects**	**Groups stratified by ethnicity**						***p***
		**White**	**Asian**	**Black**	**Chinese or other Ethnic Groups**	**Mixed**	**Not stated**	
***N***	83,414	36,331	25,148	8054	3347	1793	8741	
**Sex (women)**	42,970 (52%)	19,043 (52%)	12,784 (51%)	4277 (53%)	1703 (51%)	953 (53%)	4210 (48%)	< *0.001*
**Age at registry entry, yrs**	45 (36–48)	46 (36–61)	45 (36–57)	45 (38–55)	40 (34–50)	42 (35–52)	46 (37–61)	< *0.001*
**Age group**								< *0.001*
**30**–**39**	31,784 (38%)	13,287 (37%)	10,081 (40%)	2811 (35%)	1718 (51%)	807 (45%)	3080 (35%)	
**40**–**49**	19,852 (24%)	8047 (22%)	5767 (23%)	2597 (32%)	828 (25%)	480 (27%)	2133 (24%)	
**50**–**59**	13,313 (16%)	5527 (15%)	4602 (18%)	1172 (15%)	456 (14%)	231 (13%)	1325 (15%)	
**60**–**69**	9346 (11%)	4601 (13%)	2721 (11%)	725 (9.0%)	213 (6.4%)	137 (7.6%)	949 (11%)	
**70**–**79**	6381 (7.6%)	3190 (8.8%)	1593 (6.3%)	618 (7.7%)	98 (2.9%)	106 (5.9%)	776 (8.9%)	
**≥ 80**	2738 (3.3%)	1679 (4.6%)	384 (1.5%)	131 (1.6%)	34 (1.0%)	32 (1.8%)	478 (5.5%)	
**Areas of residence**								< *0.001*
**Bromley (BR)**	2340 (2.8%)	1554 (4.3%)	127 (0.5%)	95 (1.2%)	43 (1.3%)	38 (2.1%)	483 (5.5%)	
**Harrow (HA)**	45,688 (55%)	16,962 (47%)	19,117 (76%)	3098 (38%)	1608 (48%)	713 (40%)	4190 (48%)	
**London NW (NW)**	17,289 (21%)	7956 (22%)	3306 (13%)	2736 (34%)	1054 (31%)	590 (33%)	1647 (19%)	
**London SE (SE)**	3407 (4.1%)	2339 (6.4%)	162 (0.6%)	397 (4.9%)	116 (3.5%)	100 (5.6%)	293 (3.4%)	
**London SW (SW)**	7037 (8.4%)	4216 (12%)	328 (1.3%)	1183 (15%)	249 (7.4%)	197 (11%)	864 (3.4%)	
**Southhall (UB)**	4711 (5.6%)	1877 (5.2%)	1545 (6.1%)	248 (3.1%)	122 (3.6%)	71 (4.0%)	848 (9.7%)	
**Others**	2942 (3.5%)	1427 (3.9%)	563 (2.2%)	297 (3.7%)	155 (4.6%)	84 (4.7%)	416 (4.8%)	

*NW* Northwest, *SE* Southeast, *SW* Southwest

White participants were the oldest, whereas Mixed and Chinese/Other ethnicities included the youngest individuals; no major differences were noted in sex distribution (Table 1; Additional File 1: Fig. S4–S5). A higher proportion of non-White ethnicities lived in Harrow (62.9%), Southall (60.2%), and NW London (54%) (Table 1; Additional File 1: Fig. S6). More specifically, Asians clustered in Harrow (41.8%) and Southall (32.8%), Black groups were more common in NW (15.8%) and SW (16.8%) London, while White residents were more dispersed but dominated in Bromley, SE, and SW London.

### Cardiovascular disease risk factors reported by physicians

The unadjusted crude prevalence rates of physician-reported CVD risk factors are summarized in Table 2. Smoking was the most common, followed by obesity, diabetes, hypertension, and dyslipidemia.

**Table 2:** Physician reported rates of cardiovascular disease risk factors by ethnicity

	**Total subjects**	**Groups stratified by ethnicity**						***p***
		**White**	**Asian**	**Black**	**Chinese or other ethnic groups**	**Mixed**	**Not stated**	
***N***	83,414	36,331	25,148	8054	3347	1793	8741	
**Smokers**	6534 (7.8%)	3524 (9.7%)	1564 (6.2%)	627 (7.8%)	218 (6.5%)	152 (8.5%)	449 (5.1%)	< *0.001*
**Former smokers**	2744 (3.3%)	1747 (4.8%)	528 (2.1%)	172 (2.1%)	63 (1.9%)	43 (2.4%)	191 (2.2%)	< *0.001*
**Arterial hypertension**	3291 (3.9%)	1333 (3.7%)	1216 (4.8%)	404 (5.0%)	92 (2.7%)	64 (3.6%)	182 (2.1%)	< *0.001*
**Diabetes**	3260 (3.9%)	844 (2.3%)	1636 (6.5%)	473 (5.9%)	103 (3.1%)	68 (3.8%)	136 (1.6%)	< *0.001*
**Obesity**	3547 (4.3%)	1478 (4.1%)	1157 (4.6%)	552 (6.9%)	124 (3.7%)	76 (4.2%)	160 (1.8%)	< *0.001*
**Dyslipidemia**	1075 (1.3%)	432 (1.2%)	446 (1.8%)	95 (1.2%)	31 (0.9%)	24 (1.3%)	47 (0.5%)	< *0.001*
**Medication**								
**Lipid-lowering**	257 (0.3%)	72 (0.2%)	142 (0.6%)	25 (0.3%)	10 (0.3%)	3 (0.2%)	5 (< 0.1%)	< *0.001*
**Glucose-lowering**	140 (0.2%)	26 (< 0.1%)	85 (0.3%)	17 (0.2%)	7 (0.2%)	2 (0.1%)	3 (< 0.1%)	< *0.001*
**Blood pressure-lowering**	230 (0.3%)	64 (0.2%)	124 (0.5%)	27 (0.3%)	5 (0.1%)	2 (0.1%)	8 (< 0.1%)	< *0.001*

All values represent unadjusted, crude estimates

**Fig. 1:**
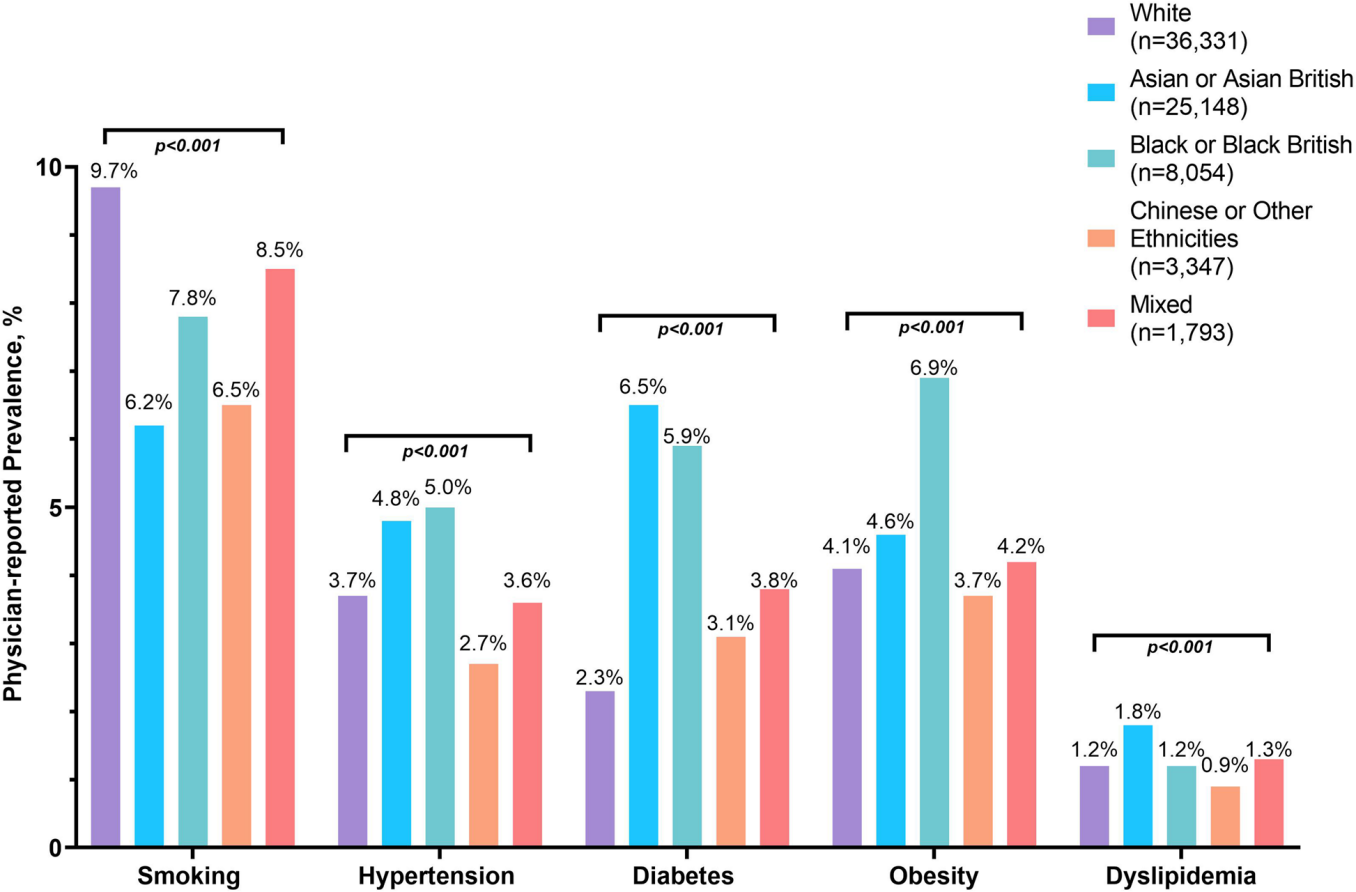
Physician-reported rates of cardiovascular disease risk factors by ethnicity. All values represent unadjusted, crude estimates

The highest prevalence of smoking was found among White individuals (9.7%). Physician-reported prevalence of obesity varied across groups, with a higher proportion in Black participants (6.9%) and a lower proportion in individuals of Chinese/Other ethnicities (3.7%). Diabetes was recorded in a higher proportion of Asian (6.5%) and Black (5.9%) individuals, whereas a smaller proportion was observed in White participants (2.3%). Similarly, hypertension was reported more often in Black (5.0%) and Asian (4.8%) groups, and less often in the Chinese/Other group (2.7%). Dyslipidemia was reported in a higher proportion of Asian individuals (1.8%), with lower proportions observed across the other ethnicities (Fig. 1).

### Body mass index, blood pressure, and lipid levels by ethnicity

Unadjusted, crude measures of BMI, BP and lipids overall and by ethnicity are shown in Table 3. Data on BMI, BP, and lipids were available in 10,093, 16,518 and 6895–8927 individuals, respectively. Among them, 31.5% had BMI ≥ 30 kg/m^2^, 48.5% had elevated BP, 44.9% had total cholesterol ≥ 5.0 mmol/L, 28.2% had elevated TG, and 25.9% had low HDL-C.

**Table 3:** Study participants’ cardiometabolic profile by ethnicity

	**Total Subjects**	**Groups stratified by ethnicities**						***p***
		**White**	**Asian**	**Black**	**Chinese or other ethnicities**	**Mixed**	**Not stated**	
**Body mass index, kg/m**^**2**^	27.2 (24.2–31.1)	27.5 (24.0–31.4)	26.7 (24.0–30.1)	28.7 (25.5–33.1)	27.4 (24.0–31.8)	27.5 (24.4–31.2)	26.9 (23.8–31.0)	< *0.001*
**Body mass index groups**								< *0.001*
** < 18.5 kg/m**^**2**^	166/10,093 (1.6%)	69/3990 (2.4%)	66/3946 (1.7%)	13/1155 (1.1%)	7/342 (2.0%)	5/212 (2.4%)	6/448 (1.3%)	
**18.5**–**25 kg/m**^**2**^	2966/10,093 (29.4%)	1192/3,990 (29.9%)	1243/3,946 (31.5%)	225/1,155 (19.5%)	97/342 (28.4%)	60/212 (28.3%)	149/448 (33.3%)	
**25**–**30 kg/m**^**2**^	3783/10,093 (37.5%)	1389/3,990 (34.8%)	1607/3,946 (40.7%)	429/1155 (37.1%)	125/342 (36.5%)	79/212 (37.3%)	154/448 (34.4%)	
** ≥ 30 kg/m**^**2**^	3178/10,093 (31.5%)	1340/3,990 (33.6%)	1030/3946 (26.1%)	488/1155 (42.3%)	113/342 (33.0%)	68/212 (32.1%)	139/448 (31.0%)	
**Systolic blood pressure, mmHg**	131 (120–142)	132 (132–143)	130 (120–140)	134 (123–145)	128 (116–139)	132 (120–145)	134 (121–144)	< *0.001*
**Diastolic blood pressure, mmHg**	80 (71–85)	80 (71–85)	79 (71–85)	80 (74–88)	77(70–84)	80 (70–87)	80 (70–86)	< *0.001*
**Blood pressure levels**								< *0.001*
**Systolic blood pressure < 130 mmHg and diastolic blood pressure < 85 mmHg**	2509/16,518 15.2%)	927/6728 (13.8%)	1123/6090 (18.4%)	222/1926 11.5%)	83/436 (19.0%)	50/328 (15.2%)	104/1010 (10.3%)	
**Systolic blood pressure 130**–**140 mmHg and/or diastolic blood pressure 85**–**90 mmHg**	6000/16,518 (36.3%)	2445/6728 (36.3%)	2251/6090 (37.0%)	684/1926 (35.5%)	167/436 (38.3%)	98/3328 (29.9%)	355/1010 (35.1%)	
**Systolic blood pressure ≥ 140 and/or diastolic blood pressure ≥ 90 mmHg**	8009/16,518 (48.5%)	3356/6728 (49.9%)	2716/6090 (44.6%)	1020/1926 (53.0%)	186/436 (42.7%)	180/328 (54.9%)	551/1010 (54.6%)	
**Total cholesterol, mmol/L**	4.80 (4.10–5.60)	4.94 (4.20–5.79)	4.70 (3.90–5.40)	4.80 (4.20–5.50)	4.90 (4.20–5.60)	5.10 (4.40–5.80)	4.90 (4.20–5.70)	< *0.001*
**Triglycerides, mmol/L**	1.27 (0.90–1.78)	1.26 (0.91–1.77)	1.34 (0.99–1.86)	0.98 (0.71–1.39)	1.30 (0.99–1.80)	1.24 (0.84–1.86)	1.30 (0.92–1.80)	< *0.001*
**High-density lipoprotein cholesterol, mmol/L**	1.30 (1.10–1.60)	1.39 (1.14–1.70)	1.25 (1.05–1.50)	1.40 (1.20–1.68)	1.30 (1.10–1.63)	1.38 (1.10–1.64)	1.30 (1.10–1.60)	< *0.001*
**Low-density lipoprotein cholesterol, mmol/L**	2.80 (2.12–3.50)	2.83 (2.20–3.60)	2.70 (2.02–3.34)	2.80 (2.24–3.54)	2.99 (2.45–3.50)	2.80 (2.20–3.55)	2.80 (2.12–3.50)	< *0.001*
**Non-high-density lipoprotein cholesterol, mmol/L**	3.36 (2.60–4.12)	3.40 (2.61–4.20)	3.30 (2.55–4.10)	3.33 (2.60–4.14)	3.40 (2.74–4.20)	3.70 (2.82–4.22)	3.40 (2.55–4.22)	*0.016*
**Total cholesterol ≥ 5.0 mmol/L**	3311/7367 (44.9%)	1410/2833 (49.8%)	1229/3093 (39.7%)	321/719 (44.6%)	112/229 (48.9%)	79/139 (56.8%)	160/354 (45.2%)	< *0.001*
**Triglycerides ≥ 1.7 mmol/L**	1945/6895 (28.2%)	741/2598 (28.5%)	889/2875 (30.9%)	104/720 (14.4%)	58/201 (28.9%	41/141(29.1%)	112/360 (31.1%)	< *0.001*
**High-density lipoprotein cholesterol < 1.0 mmol/L for males or < 1.3 mmol/L for females**	2311/8927 (25.9%)	723/3362 (21.5%)	1193/3776 (31.6%)	179/920 (19.5%)	69/272 (25.4%)	43/167 (25.7%)	104/430 (24.2%)	< *0.001*
**Low-density lipoprotein cholesterol, ≥ 4.9 mmol/L**	183/7023 (2.6%)	85/2712 (3.1%)	58/2869 (2.0%)	24/726 (3.3%)	3/194 (1.5%)	6/157 (3.8%)	7/365 (1.9%)	*0.014*

All values represent unadjusted, crude estimates

**Fig. 2:**
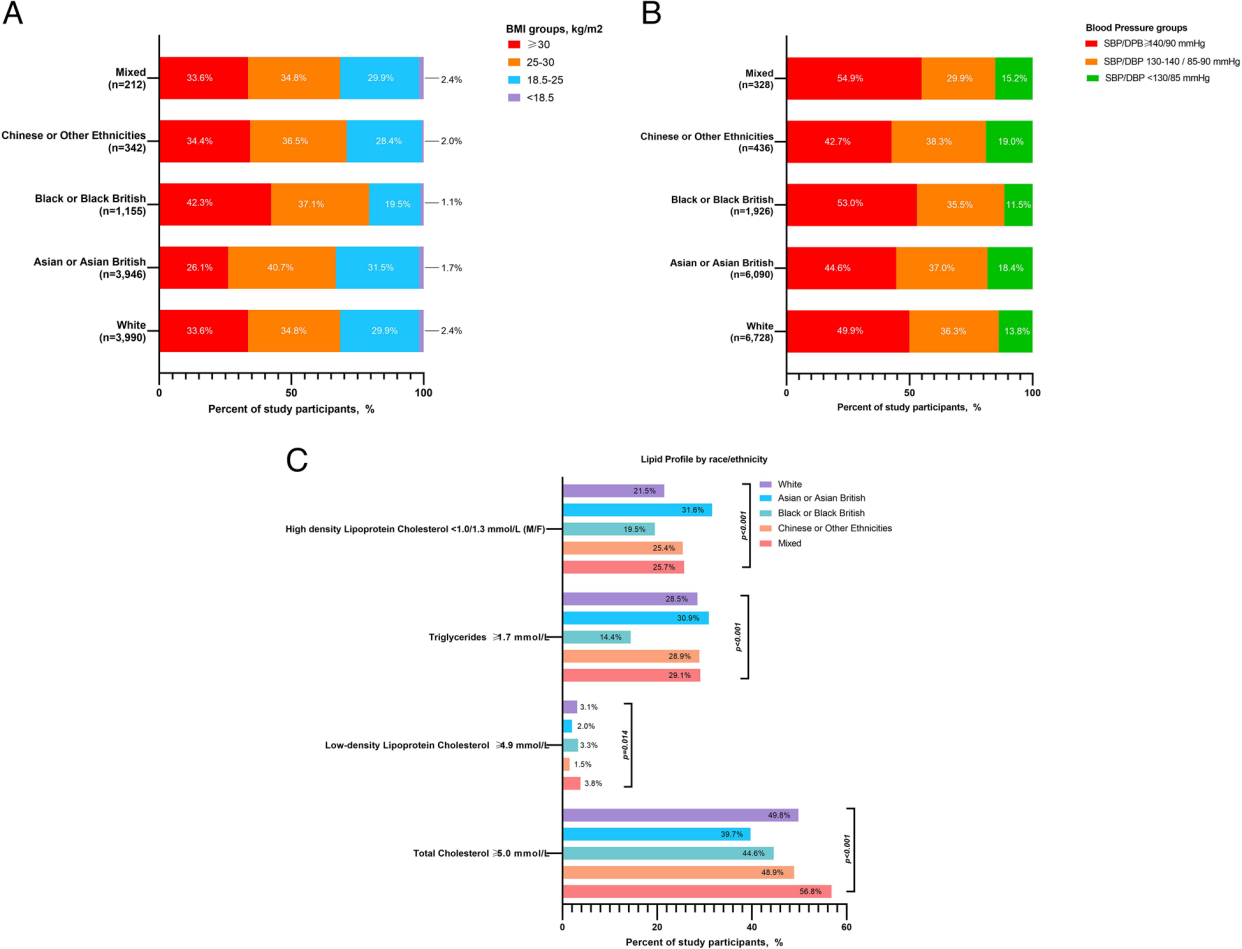
Body mass index, blood pressure and lipid levels by ethnicity. All values represent unadjusted, crude estimates

The overall median BMI was 27.2 kg/m^2^ (24.2–31.1) and varied across ethnic groups, with the highest levels recorded in Black participants and the lowest in those of Asian ethnicity (Table 3). Obesity was more frequently documented in Black participants (42.3%) and less often in Asian (26.1%) individuals (Fig. 2A).

Systolic and diastolic BP levels differed by ethnicity, with the highest median levels noted in Black participants and the lowest in Chinese/Other participants (Table 3). Elevated BP was observed in higher proportions of Mixed (54.9%) and Black (53%) groups, with a lower proportion recorded in individuals classified as Chinese/Other (42.7%) (Fig. 2B).

Variation was also observed in lipids across ethnic groups (Table 3). Total cholesterol ≥ 5.0 mmol/L was observed in higher proportions of Mixed (56.8%) and White (49.8%) participants, with lower proportions recorded in Asian ethnic groups (39.7%). Elevated TG and low HDL-C were documented in a higher proportion of Asian participants (30.9% and 31.6%, respectively), while these rates were less common in Black individuals (14.4% and 19.5%, respectively) (Fig. 2C).

### Body mass index, blood pressure, and lipid levels by age

**Fig. 3:**
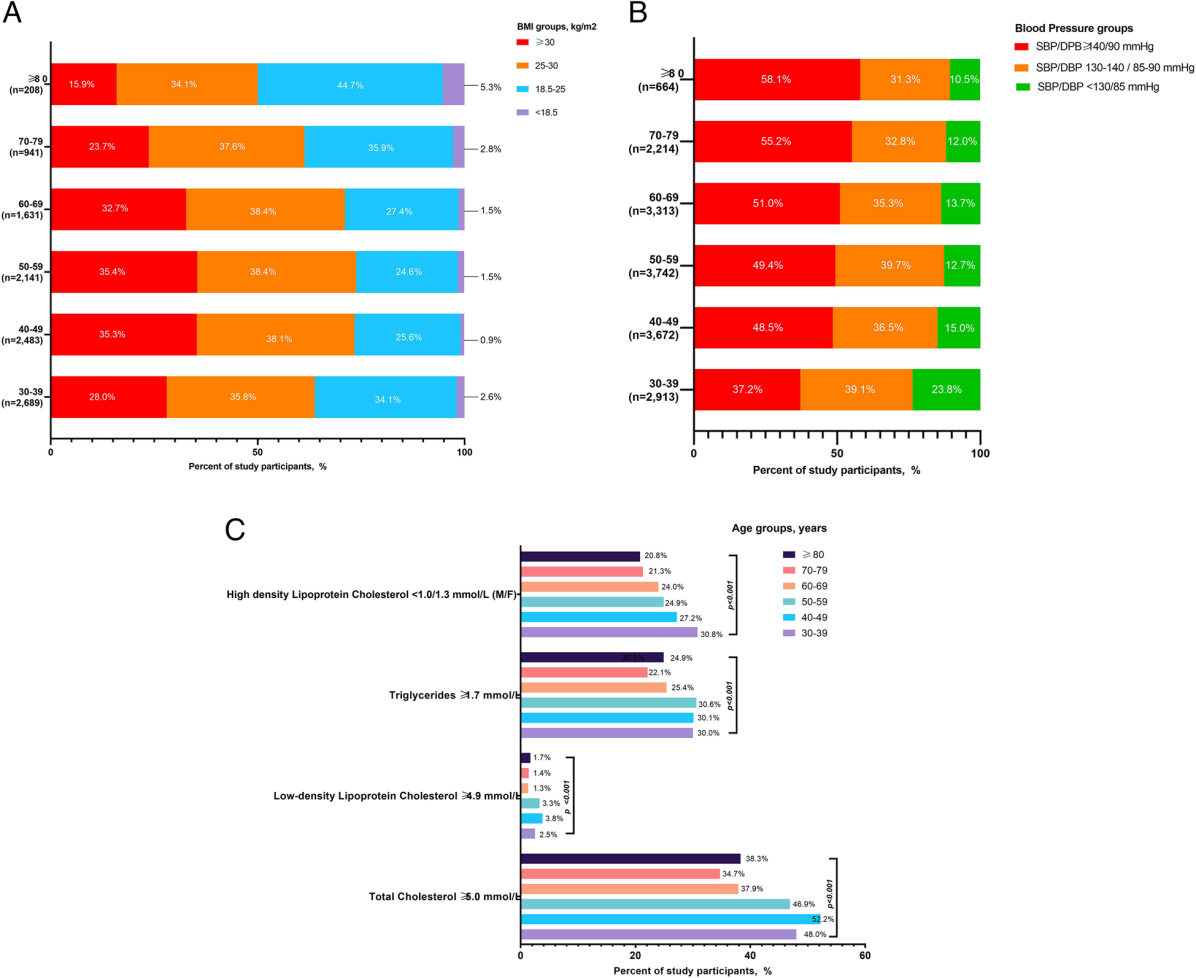
Body mass index, blood pressure and lipid levels by age group. All values represent unadjusted, crude estimates

Age-stratified analyses revealed substantial variations in BMI, BP and lipid levels across the lifespan (Additional File 1: Table 1). Median BMI levels peaked among individuals aged 50–59 years and declined in those ≥ 80 years. Obesity prevalence increased from 28% in the participants aged 30–39 years, was higher among those aged 40–59 years (35.3–35.4%) and lower (15.9%) in the ≥ 80-year group (Fig. 3A).

Median systolic BP was higher in the older age groups, while median diastolic BP was lower in the younger age groups (Additional File 1: Table 1). The prevalence of elevated BP increased with increasing age group, from 37.2% in the 30–39 group to 58.1% in those aged ≥ 80 years (Fig. 3B).

Total cholesterol was highest among individuals aged 30–49 years and declined in older age groups (Additional File 1: Table 1). Elevated total cholesterol was more frequent in the 30–49 group (48–52.2%) and less common (38.3%) in those ≥ 80 years (Fig. 3C). The prevalence of elevated TG levels followed a similar pattern, peaking in participants aged 50–59 years (30.6%) and declining thereafter (24.9% in ≥ 80 years). Prevalence of low HDL-C was higher in younger participants, particularly in the 30–39 group (30.8%) and progressively decreased in the higher age categories (20.8% in ≥ 80 years; Fig. 3C).

### Body mass index, blood pressure, and lipid levels by area of residence

BMI, BP, and lipid levels varied moderately across residential areas (Additional File 1: Fig. S7 and Table 2). Obesity prevalence ranged from 36.9% in Bromley to 30.1% in Harrow. No major differences in BP levels were noted across different London areas. The prevalence of hypercholesterolemia ranged from 51.2% in Bromley to 41.4% in London SE. Elevated TG and low HDL-C values were more frequently recorded in Southhall (31.3% and 37.6%, respectively).

## Discussion

In this large EHR-based cohort of 83,414 apparently healthy individuals attending London primary care practices between 2009 and 2020, we identified a high burden of CVD risk factors. Nearly half of participants had elevated BP or hypercholesterolemia, and one in three were living with obesity, or had elevated TG, while the corresponding rates of physician-recorded diagnoses were considerably lower, meaning that these highly prevalent conditions may be underdiagnosed in clinical practice, hence with the potential implications of undertreatment. We also observed ethnic and age-related differences in the prevalence of modifiable CVD risk factors. Diabetes was observed in higher proportions of Black and Asian individuals, while smoking was more frequently reported in the White group. Black individuals tended towards higher proportions of obesity and elevated BP, whereas Asian individuals tended to exhibit a more atherogenic lipid profile. In addition, most CVD risk factors were already evident in individuals as early as their 30 s–40 s. Taken together, this evidence reinforces the need for tailored preventive interventions considering ethnicity diversity and beginning in early adulthood.

Consistent evidence over the past decade supports the contribution of ethnic differences to the prevalence of CVD risk factors in UK populations [5, 6, 10–14, 16, 31, 32]. In London-based cohorts (Whitehall II, SABRE), South Asian groups (predominantly from the Indian subcontinent) consistently showed a higher prevalence of diabetes and more atherogenic lipid profiles consistent with insulin resistant traits, while Black/Afro-Caribbean groups had a higher prevalence of hypertension and obesity, but less adverse lipid profiles compared with White counterparts [6, 8]. National data from the Health Survey for England (HSE) and UK Biobank likewise reported higher prevalence of hypertension, diabetes and obesity in Black and South Asian groups (Indian, Pakistani, Bangladeshi or other Asian), adverse lipids particularly in South Asians, and lower levels of these conditions with more favourable lipid profiles in Chinese ethnic groups [10, 11, 13, 32]. Similar patterns have recently been confirmed in large EHR-based cohorts across multiple UK regions [14, 16]. Our study, conducted between 2009 and 2020 in London, demonstrates broadly similar associations by ethnic groups further providing contemporary, real-world confirmation that disparities in risk factors across ethnic groups, previously identified, continue to persist. More generally, these findings underscore the relevance of prevention strategies that consider the evolving ethnic landscape in any region/country to better deliver appropriate prevention strategies.

Understanding the reasons behind the varying prevalence of CVD risk factors across ethnicities is a crucial next step for shaping effective public health strategies. Minority ethnic groups have been reported to differ from White individuals in dietary and sleep habits, to engage in less physical activity, and to have lower cardiorespiratory fitness [33]. Beyond lifestyle behaviours, broader sociodemographic and environmental factors, including higher levels of deprivation, differences in the building environment, weaker community and social support, inequalities in socioeconomic status, education, and access to healthcare services, are also important determinants of cardiovascular and overall health [33]. Although such variables are not routinely collected in EHRs and were therefore not considered in our study, analyses from the aforementioned cohorts indicate that, even after adjustment for sociodemographic, lifestyle, environmental, and clinical factors, South Asian and Black individuals remain disproportionately affected by diabetes [7, 16]. Moreover, South Asians continue to experience an increased risk of CVD that is not fully captured by traditional risk calculators, whereas Black populations, despite a lower overall CVD risk, appear to be more prone to stroke [8, 11, 13, 14, 32, 34]. That said, the attributable risk for CVD varies by ethnicity, with diabetes and obesity being key contributors to CVD risk among Asian and Black populations [5, 8, 9, 13, 35, 36]. Taken together, these observations highlight that CVD risk at the population level is multifactorial and strongly patterned by ethnicity, underscoring the need for prevention strategies that move beyond a “one-size-fits-all” approach and are tailored to ethnicity-specific risk profiles and social determinants.

According to the Global Cardiovascular Risk Consortium, five traditional modifiable risk factors (BMI, systolic BP, non–HDL-C, smoking, and diabetes) measured at midlife account for half of all cases of CVD globally, with variations across different populations [24]. Likewise, previous studies in the UK have shown that midlife measurement of these risk factors does not fully explain the ethnic differences observed in CVD incidence and mortality [8, 13]. In our study, the prevalence of CVD risk factors was already increasing from as early as the third decade of life. Taken together, this evidence highlights the importance of cumulative exposure to risk factors over time and underscores the need for earlier, ethnicity-informed prevention strategies that begin well before midlife.

NHS Health Check Programme in the UK is an example of a public health intervention aiming to tackle CVD and health ethnic inequalities. Although completing its first decade in 2020, with coverage averaging 1 million annually, its effectiveness has been challenged by some researchers and clinicians, and there is ongoing debate about whether mass or targeted cardiovascular screenings are more efficient and cost-effective for certain ethnic groups [37, 38]. According to evidence derived from studies conducted in specific areas in the UK, the NHS Health Check Programme increased the detection of CVD risk factors, but the latter was inversely associated with deprivation [38–40]. Furthermore, its uptake varied between population subgroups depending on the participants’ sex, age, deprivation, and race or ethnicity, while corresponding increases in evidence-based interventions were modest [38, 39]. On the other hand, others have speculated that opportunistic CVD risk assessment might be superior to population health checks and raised concerns that the programme could inadvertently widen health inequalities [19–21]. Although we did not specifically assess NHS Health Check uptake, our study of apparently healthy individuals undergoing primary prevention CVD risk assessments, with only ~ 2% recorded to complete an NHS Health Check, showed high rates of suboptimal BMI, BP, and lipid levels, despite the low prevalence of physician-reported diagnoses of obesity, hypertension, and dyslipidemia. For context, physician-reported obesity prevalence in the total study population was only 4.3%, whereas objective BMI-derived prevalence was 31.5%. Similarly, physician-reported hypertension was 3.9%, compared with a BP-derived prevalence of elevated blood pressure of 48.5%. These findings suggest that the population health checks, like NHS Health Check Programme, may play a valuable role in the early identification of CVD-related risk factors and comorbidities in the general population. However, whether early detection consistently translates into effective diagnosis and intervention across diverse populations remains uncertain.

The substantial discrepancy between physician-reported disease diagnoses and objectively measured prevalence rates represents a critical finding suggesting widespread underdiagnosis and/or incomplete documentation in primary care. This diagnostic gap may reflect interconnected systemic barriers, including EHR workflows that fail to translate measured abnormalities into documented diagnoses, time constraints that limit clinical recognition and physician inertia [41–43]. The consequences extend beyond documentation, since formal diagnosis serves as a necessary trigger for evidence-based clinical management. For instance, studies demonstrate that individuals with formally recorded diagnoses of obesity and hypertension are significantly more likely to receive intervention and follow-up than those with measured but undocumented relative diagnoses [42, 43]. Notably, a delayed diagnosis of hypertension by more than 1 year is associated with a 29% increased risk of major adverse cardiovascular events and substantially lower treatment prescription rates, indicating that the diagnostic gap directly translates to increased patient morbidity [43]. At the population level, underdiagnosis distorts disease burden estimates used for public health planning and resource allocation, disproportionately affects disadvantaged populations already experiencing higher disease burden, and undermines the effectiveness of screening programs by preventing translation of measured abnormalities into clinical management [44]. Taken together, these findings underscore that improving the timely recognition and recording of common cardiometabolic conditions is not merely an issue of data quality but a critical target for reducing preventable cardiovascular events, addressing inequalities in care, and maximizing the population impact of primary care–based screening initiatives.

There are similarities and significant differences between the present study and prior relevant large-scale studies that merit discussion. The Whitehall II study, conducted between 1985 and 1988, focused on civil servants and lacked ethnic diversity (only 9.4% non-White individuals) and general population representation [6]. The Southall and Brent Revisited (SABRE) study provides valuable multiethnic insights but was geographically restricted and based on data collected primarily in earlier decades (1988–91 and 2008–2013) [7–9]. The HSE, while nationally representative, was a survey conducted in 1999 and 2004 and included relatively small samples of minority ethnic groups [10, 11]. UK Biobank, although extensive, is limited by healthy volunteer bias and under-representation of ethnic minorities, reducing generalizability to the broader population [12, 13]. Unlike prior EHR analyses with larger datasets including up to two million participants [14–16], our study focused on data from the most recent decade (2009–2020) in London and was restricted to GP, thereby capturing mostly primary prevention individuals. This approach yielded a more ethnically diverse population and provides an updated perspective on health inequalities in the contemporary era.

### Study limitations

There are some limitations that should be acknowledged. The cross-sectional design of the study precluded any investigation of longitudinal changes in CVD risk factors. A major limitation in our study was the lack of clinical information for more than 465,000 of the 607,000 registered individuals (> 75%). The restriction of our analyses to persons without CVD could have led to the underrepresentation of the elderly and other groups at high risk for disparities (Asian or Black patients with premature CVD). Importantly, this restriction may also have resulted in an underestimation of the overall burden of risk factors, since individuals with such risk factors are more likely to have developed CVD and were therefore excluded from the study. However, in general population studies, risk factors are not always recorded at the time of healthcare consultation for other ailments. It should be noted that in UK Biobank (age 40 to 69 years) approximately 5.5% of those invited attended for full assessment [45], which is less than the ~ 25% with available data in the present study. Ethnicity and postcode of residence were self-reported, which may introduce misclassification bias.

In line with previous UK studies utilizing health records [14, 16, 46], the use of only five aggregated ethnicity categories in our study may have obscured important heterogeneity within smaller subgroups (e.g. Chinese, Pakistani, Bangladeshi, Indian), while inclusion of the “not stated” group may have led to an underestimation of the observed differences between ethnic groups. That said, information on migration-related factors, such as country of birth and first- versus second-generation status, was not available, limiting our ability to further contextualize ethnic differences in CVD risk. Furthermore, we applied a uniform BMI ≥ 30 kg/m^2^ threshold, which may have underestimated obesity prevalence in certain populations, particularly among Asian and Chinese groups, for whom lower BMI cut-offs are recommended. Data on important CVD risk factors and social determinants (e.g. physical activity, education, deprivation index) as well as family history and diet are not routinely captured in EHRs and were therefore sparsely available, which may have led to residual confounding. Potential sources of bias include sample selection and inherent limitations of EHR data, such as imprecise variable definitions, variability in measurement frequency, and incomplete or inaccurate records. Moreover, relying solely on crude prevalence estimates, without age-standardized comparisons or formal ethnicity–age interaction analyses, limits the interpretability and generalizability of the findings and cannot provide information on why the differences were observed. These should be investigated in future studies. Finally, the participating practices may not be fully representative of all London or UK practices, and detailed practice-level characteristics (e.g. list size, deprivation scores, ethnic composition) were not available.

## Conclusions

The results of this large-scale, contemporary analysis including a markedly ethnically diverse population in different areas of London show ethnic and age-related variation in the prevalence of modifiable CVD risk factors in asymptomatic individuals in primary care. Given the descriptive nature of the study, these findings cannot provide etiological insights but may reflect real-world gaps and disparities in risk factor identification and management. That said, the higher burden of obesity, hypertension, diabetes, and dyslipidemia observed in specific ethnic groups, particularly among Black and Asian populations, alongside their presence as early as the third decade of life, supports an appraisal of early implementation of targeted prevention strategies in specific populations in primary care to reduce health inequalities and potentially improve cardiovascular outcomes in diverse populations.

## Supplementary Information

Additional file 1. Fig S1–Flow chart of study participant selection and data extraction. Fig S2–Residential areas of study participants. Fig S3–Study population (*n* = 83,414) by ethnicity. Fig S4–Median age by ethnicity. Fig S5–Age groups by ethnicity. Fig S6–Distribution of ethnicities by area of residence. Fig. S7-Body mass index, blood pressure and lipid levels by area of residence.

## Data Availability

The datasets generated and/or analyzed during the current study are not publicly available due to privacy restrictions.

## Abbreviations

BMI: Body mass index
BP: Blood pressure
BR: Bromley (a residential area)
BDAU: Big Data Analytics Unit
CHD: Coronary heart disease
CVD: Cardiovascular disease
DBP: Diastolic blood pressure
EHR: Electronic health records
EMIS: Egton Medical Information Systems
GP: General practice
HA: Harrow (a residential area)
HDL-C: High-density lipoprotein cholesterol
HSE: Health Surveys for England
IQR: Interquartile ranges
LDL-C: Low-density lipoprotein cholesterol
NHS: National Health System
non-HDL-C: Non-high-density lipoprotein cholesterol
NW: Northwest London (a residential area)
SABRE: Southall And Brent Revisited (a study)
SBP: Systolic blood pressure
SE: Southeast London (a residential area)
SNOMED CT: Systematized Nomenclature of Medicine—Clinical Terms
SW: Southwest London (a residential area)
TG: Triglycerides
UB: Southall (a residential area)
UK: United Kingdom
WHO: World Health Organization
