# Supplementary material for: Ethnic differences in the burden of cardiovascular disease risk factors among adult residents of London: the TOGETHER study
Source: BMC Med. 2026 Mar 3;24:220. doi: 10.1186/s12916-026-04739-6 (PMC13067566; doi:10.1186/s12916-026-04739-6)
Supplement: Supplementary file 1 — Additional file 1. Fig S1–Flow chart of study participant selection and data extraction. Fig S2–Residential areas of study participants. Fig S3–Study population (n = 83,414) by ethnicity. Fig S4–Median age by ethnicity. Fig S5–Age groups by ethnicity. Fig S6–Distribution of ethnicities by area of residence. Fig. S7-Body mass index, blood pressure and lipid levels by area of residence. [file 12916_2026_4739_MOESM1_ESM.docx]

**Supplementary Appendix to**

**Ethnic Differences in the Burden of Cardiovascular Disease Risk Factors Among Adult Residents of London: The TOGETHER Study**

Fotios Barkas, Malo Dirou, Kanika I. Dharmayat, Mazidi Mahsa, Antonio J. Vallejo-Vaz, Christophe A.T. Stevens, Amany Elshorbagy, Kausik K. Ray

**Tables of Contents**

[**Fig. S1.** Flow chart for selection of eligible study participants and data extraction process 3](#_Toc221450374)

[**Fig. S2**. Residential areas of study participants. 5](#_Toc221450375)

[**Fig. S3.** Study population (n = 83,414) by ethnicity 6](#_Toc221450376)

[**Fig. S4.** Median age by ethnicity 7](#_Toc221450377)

[**Fig. S5**. Age group by ethnicity 8](#_Toc221450378)

[**Fig. S6.** Distribution of ethnicities by area of residence 9](#_Toc221450379)

[**Fig. S7.** Body mass index, blood pressure and lipid levels by area of residence 10](#_Toc221450380)

[**Table 1.** Body mass index, blood pressure and lipid levels by age group 13](#_Toc221450381)

[**Table 2.** Body mass index, blood pressure and lipid levels by area of residence 16](#_Toc221450382)

# **Fig. S1.** Flow chart for selection of eligible study participants and data extraction process

Total individuals registered with participating GPs (n = 607,327)

Excluded due to having no clinical information available (n = 465,663)

Individuals with at least one clinical observation (n = 141,664)

Excluded due to

- Irrelevant clinical information (n=55,825)
- Age <30 / >90 yrs old or CVD presence (n = 2,425)

Total eligible study participants (n= 83,414)

Clinical information was obtained from EMIS Web/EMIS LV electronic health records and extracted into three core datasets: Patient, Observation, and Clinical Codes dataframes. These datasets were subsequently merged. Overall, 141,664 unique patients had at least one recorded clinical description, contributing 3,733,265 observations from which 25,759 unique clinical descriptions were identified.

To restrict the data to cardiometabolic risk factors and cardiovascular disease, we applied a pre-specified list of cardiovascular-related keywords: “Systolic”, “systolic”, “pressure”, “Pressure”, “Triglyceride”, “LDL”, “HDL”, “cholesterol”, “Cholesterol”, “Lipid”, “lipid”, “triglyceride”, “lipoprotein”, “lipoproteins”, “Glucose”, “glucose”, “Obesity”, “obesity”, “Alcohol”, “alcohol”, “hypertension”, “Hypertension”, “Diabetes”, “diabetes”, “Body mass index”, “body mass index”, “Smoker”, “smoker”, “hypercholesterol”, “Hypercholesterol”, “lipida”, “Transient”, “transient”, “cerebrovascular”, “Cerebrovascular”, “overweight”, “peripheral”, “Peripheral”, “Metabolic”, “cardiovascular”, “Cardiovascular”, “coronary”, “Coronary”, “stroke”, “Stroke”, “myocardial”, “Myocardial”. This yielded 655,388 CVD-related observations corresponding to 1,162 unique clinical descriptions in 85,839 patients.

These 1,162 CVD-related clinical descriptions were manually reviewed and grouped into a reduced set of harmonised terms reflecting major cardiovascular risk factors and diagnoses (e.g. blood pressure/hypertension, lipids/dyslipidaemia, glucose/diabetes, BMI/obesity, smoking status, cardiovascular disease diagnoses, family history). The recoded terms were then used to derive binary indicators and continuous variables for each risk factor, using both coded diagnoses and, where relevant, associated numeric values (e.g. blood pressure, body mass index and lipid levels). Finally, after excluding age <30 / >90 yrs old or CVD presence upon their first recorded visit (n = 2,425), the total sample comprised of 83,414 eligible participants with at least one relevant clinical description.

# **Fig. S2**. Residential areas of study participants.

**
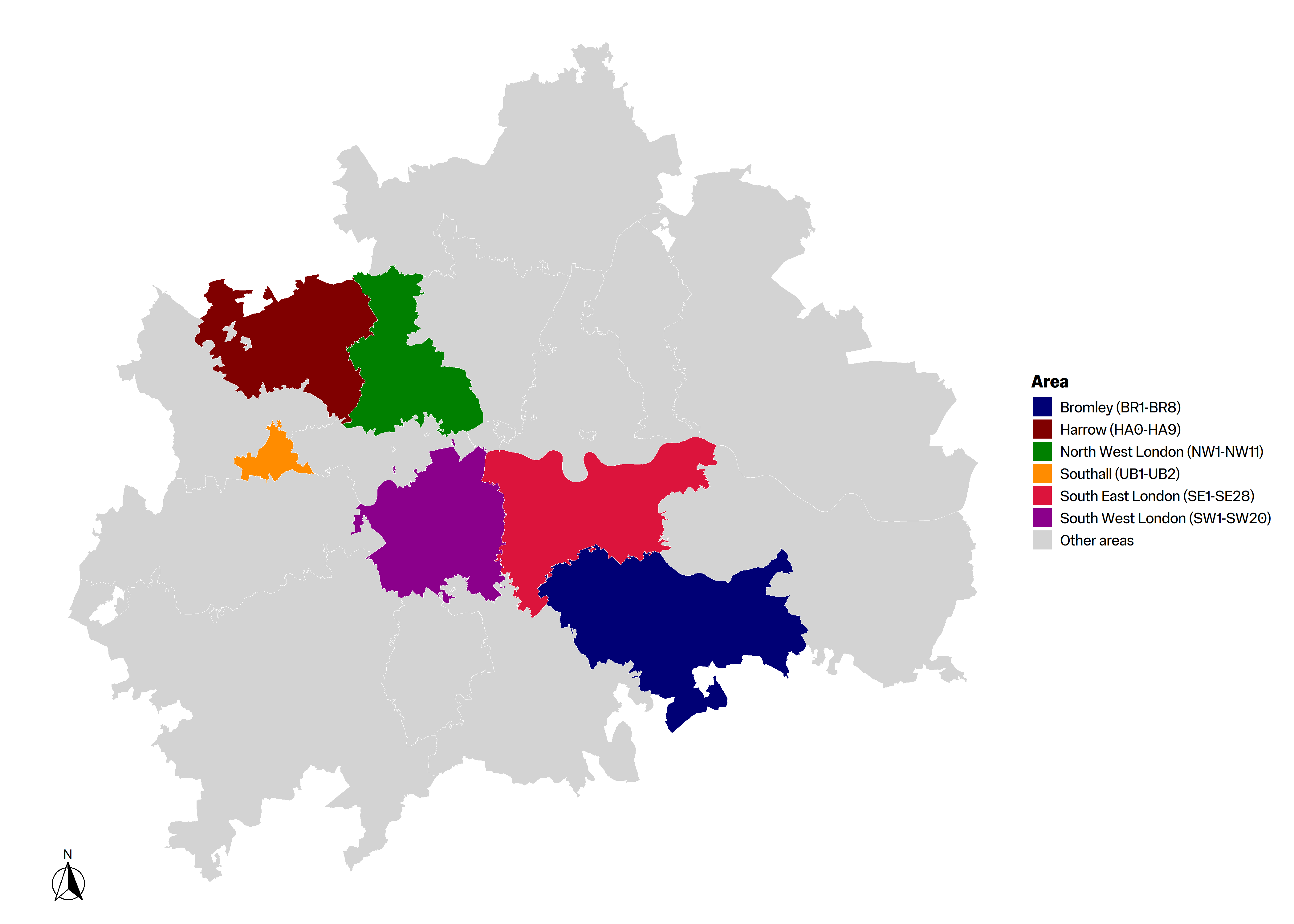
**

Areas were defined according to self-reported postcodes. Regions with ≥2,000 participants were analysed separately: Bromley (BR), Harrow (HA), Northwest London (NW), Southeast London (SE), Southwest London (SW), and Southall (UB). All other postcodes with <2,000 participants were grouped as “other areas.”

# **Fig. S3.** Study population (n = 83,414) by ethnicity


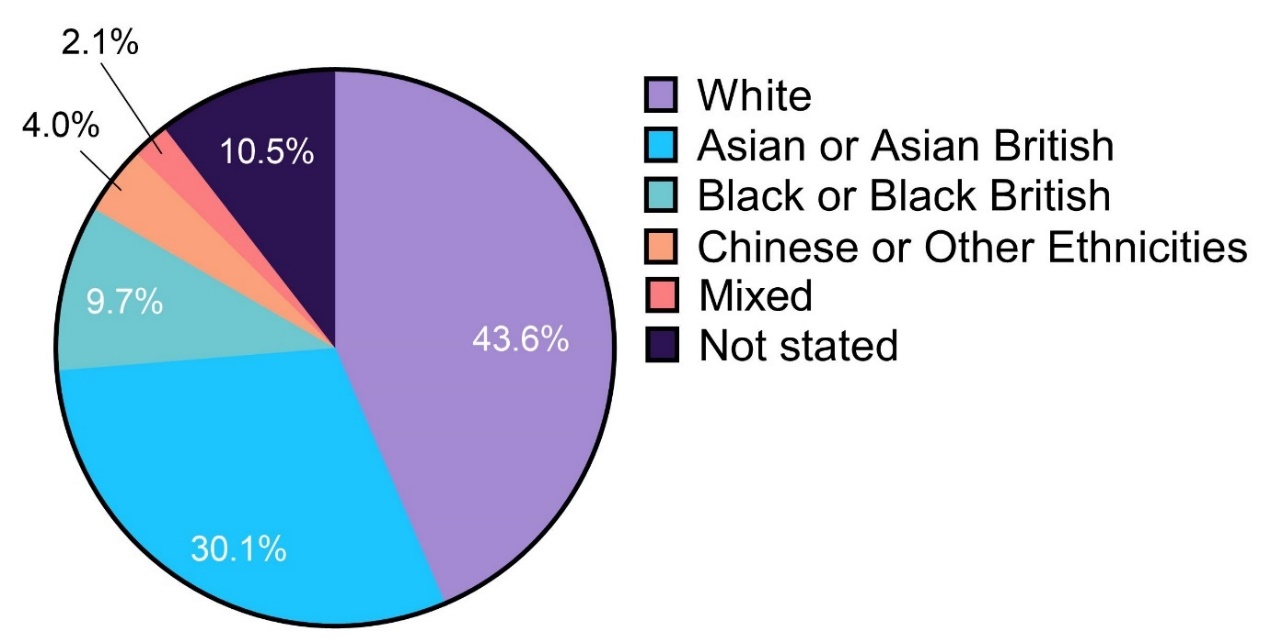


# **Fig. S4.** Median age by ethnicity


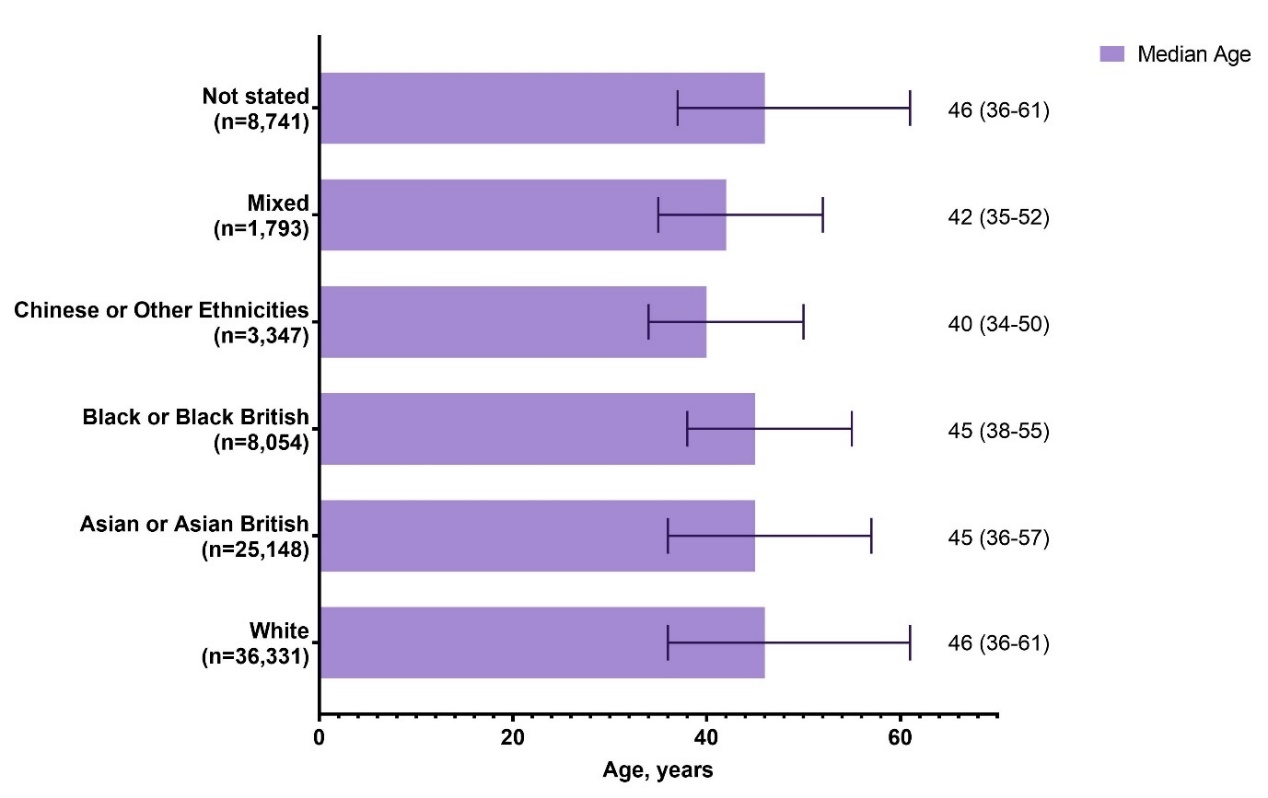


# **Fig. S5**. Age group by ethnicity

**
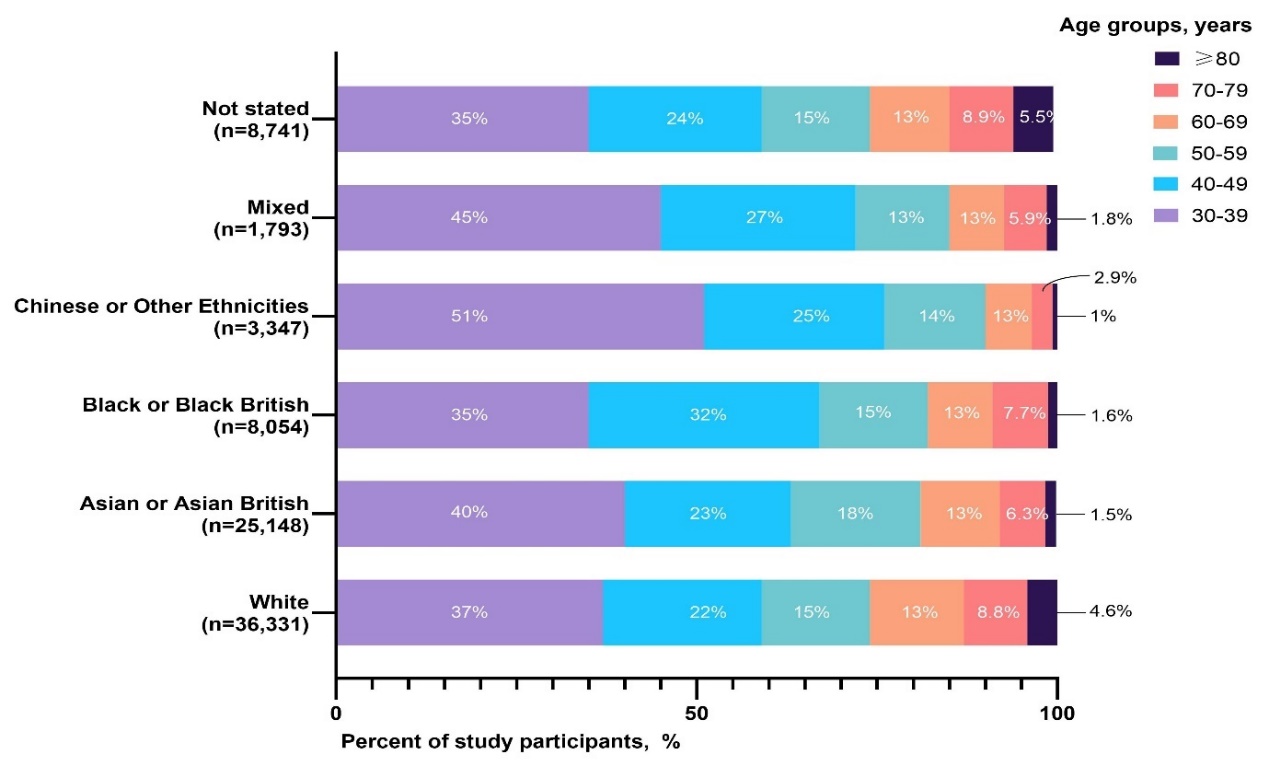
**

# **Fig. S6.** Distribution of ethnicities by area of residence


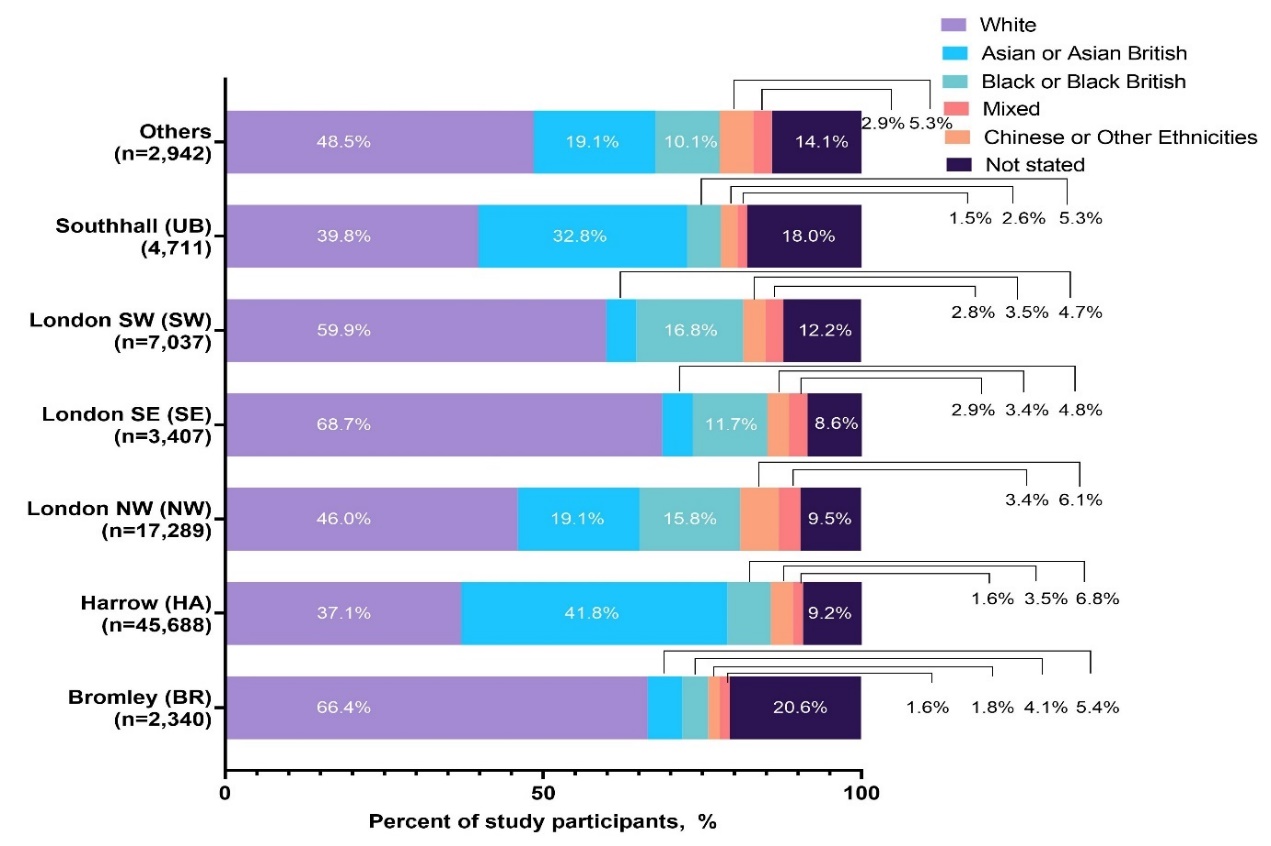


**Fig. S7.** Body mass index, blood pressure and lipid levels by area of residence
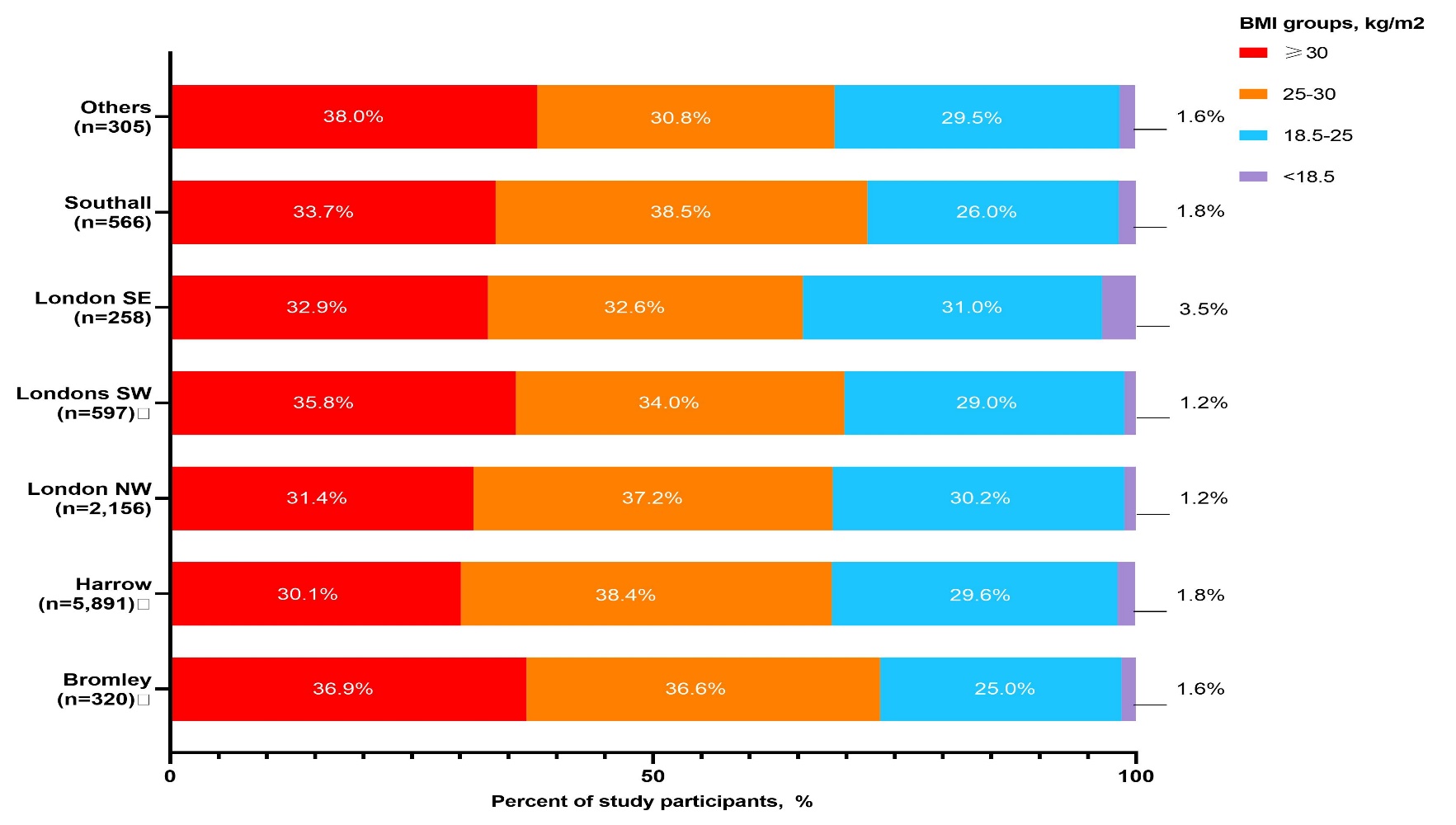


Α. Body mass index by area of residence


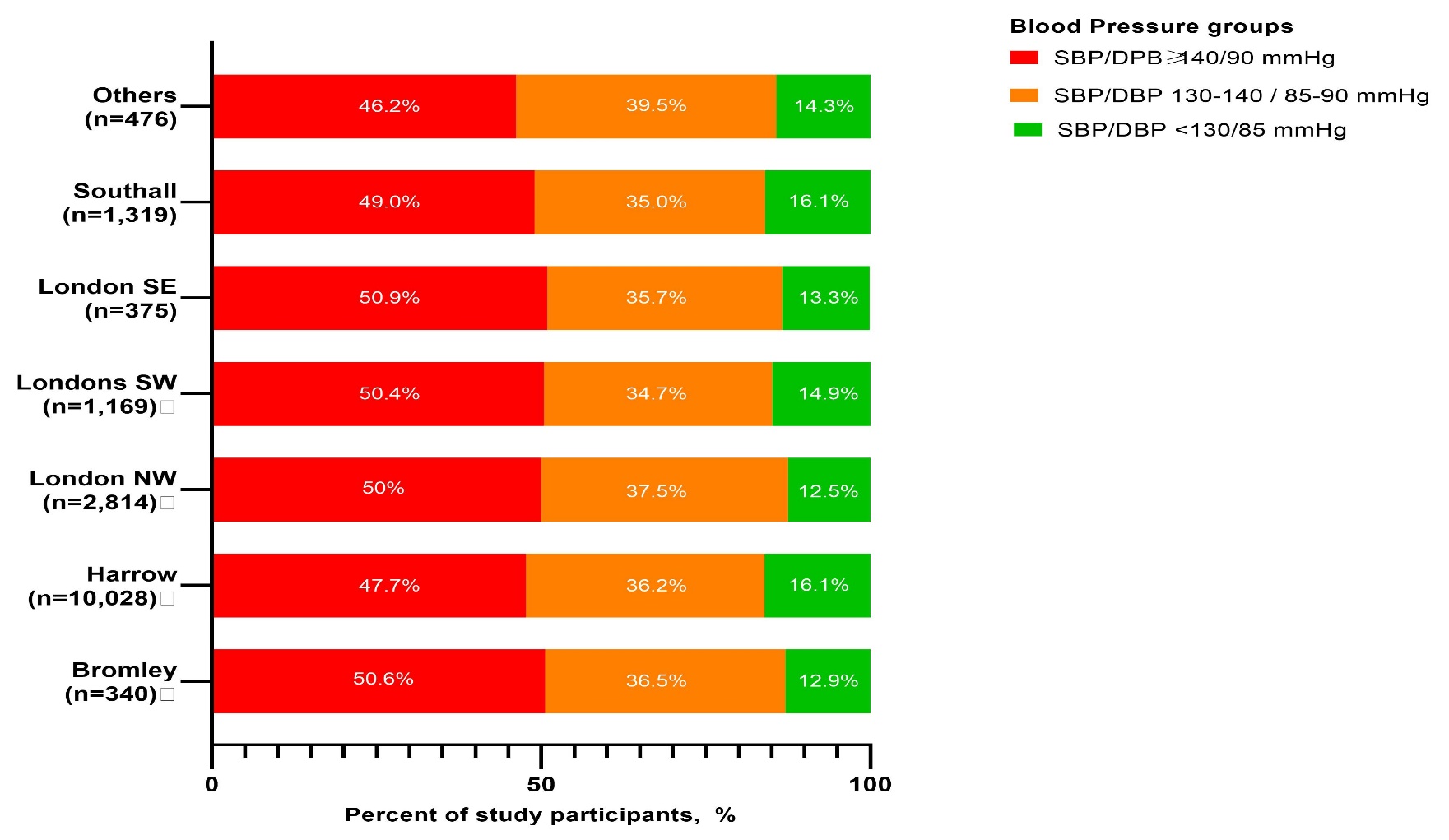


B. Blood pressure by area of residence


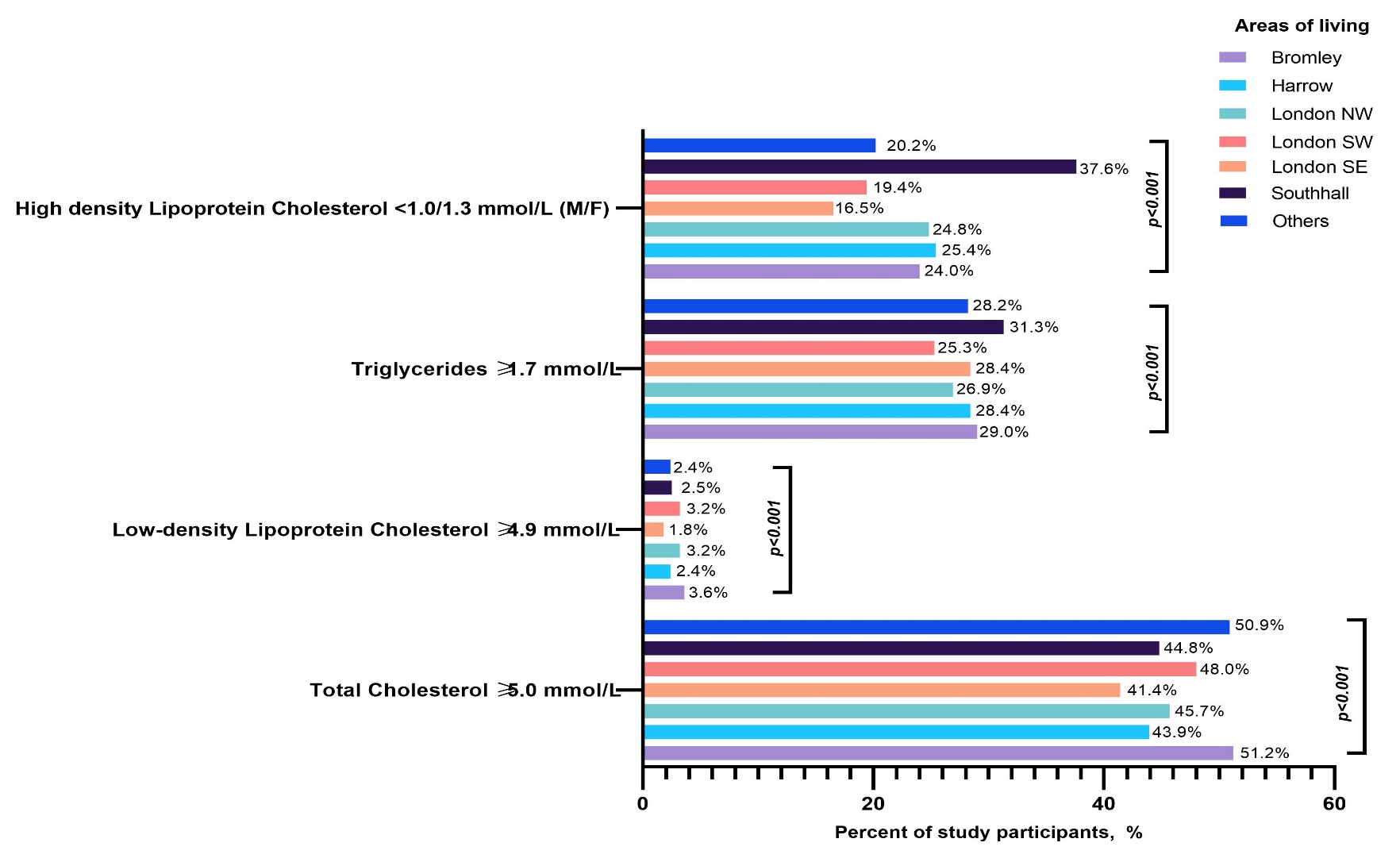


C. Lipids by area of residence

# **Table 1.** Body mass index, blood pressure and lipid levels by age group

|  | **Total subjects** | **Groups stratified by age groups** | | | | | | |
| --- | --- | --- | --- | --- | --- | --- | --- | --- |
|  |  | **30-39** | **40-49** | **50-59** | **60-69** | **70-79** | **≥80** | ***P*** |
| **Body Mass Index, kg/m^2^** | 27.2  (24.2-31.1) | 26.6  (23.6-30.5) | 27.7  (24.7-31.7) | 27.9  (24.8-31.7) | 27.5  (24.4-31.2) | 26.3  (23.4-29.7) | 25.0  (22.0-28.4) | *<0.001* |
| **Body Mass Index groups** |  |  |  |  |  |  |  | *<0.001* |
| **<18.5 kg/m^2^** | 166/10,093 | 69/2,689 | 22/2,483 | 33/2,141 | 24/1,631 | 26/941 | 11/208 |  |
|  | (1.6%) | (2.6%) | (0.9%) | (1.5%) | (1.5%) | (2.8%) | (5.3%) |  |
| **18.5-25 kg/m^2^** | 2,966/10,093 | 923/2,689 | 636/2,483 | 527/2,141 | 447/1,631 | 338/941 | 93/208 |  |
|  | (29.4%) | (34.3%) | (25.6%) | (24.6%) | (27.4%) | (35.9%) | (44.7%) |  |
| **25-30 kg/m^2^** | 3,783/10,093 | 963/2,689 | 946/2,483 | 823/2,141 | 626/1,631 | 354/941 | 71/208 |  |
|  | (37.5%) | (35.8%) | (38.1%) | (38.4%) | (38.4%) | (37.6%) | (34.1%) |  |
| **≥30 kg/m^2^** | 3,178/10,093 | 753/2,689 | 877/2,483 | 758/2,141 | 534/1,631 | 223/941 | 33/208 |  |
|  | (31.5%) | (28.0%) | (35.3%) | (35.4%) | (32.7%) | (23.7%) | (15.9%) |  |
| **Systolic Blood Pressure, mmHg** | 131  (120-142) | 122  (112-133) | 130  (120-140) | 135  (125-145) | 136  (126-146) | 138  (127-149) | 138  (126-150) | *<0.001* |
| **Diastolic Blood Pressure, mmHg** | 80  (71-85) | 78  (70-85) | 80  (75-89) | 80  (74-86) | 79  (70-84) | 75  (69-80) | 74  (67-80) | *<0.001* |
| **Blood pressure levels** |  |  |  |  |  |  |  | *<0.001* |
| **Systolic Blood Pressure <130 mmHg and Diastolic Blood Pressure <85 mmHg** | 2,509/16,518  (15.2%) | 692/2,913  (23.8%) | 552/3,672  (15.0%) | 475/3,742  (12.7%) | 454/3,313 (13.7%) | 266/2,214  (12.0%) | 70/664  (10.5%) |  |
| **Systolic Blood Pressure 130-140 mmHg and/or Diastolic Blood Pressure 85-90 mmHg** | 6,000/16,518  (36.3%) | 1,138/2,913  (39.1%) | 1,339/3,672  (36.5%) | 1,418/3,742  (37.9%) | 1,171/3,313  (35.3%) | 726/2,214  (32.8%) | 208/664  (31.3%) |  |
| **Systolic Blood Pressure ≥140 and/or Diastolic Blood Pressure ≥90 mmHg** | 8,009/16,518  (48.5%) | 1,083/2,913  (37.2%) | 1,781/3,672  (48.5%) | 1,849/3,742  (49.4%) | 1,688/3,313  (51.0%) | 1,222/2,214  (55.2%) | 386/664  (58.1%) |  |
| **Total cholesterol, mmol/L** | 4.80  (4.10-5.60) | 4.90  (4.30-5.60) | 5.00  (4.30-5.70) | 4.90  (4.01-5.70) | 4.65  (3.90-5.40) | 4.50  (3.80-5.30) | 4.50  (3.82-5.37) | *<0.001* |
| **Triglycerides, mmol/L** | 1.27  (0.90-1.78) | 1.26  (0.89-1.84) | 1.30  (0.90-1.83) | 1.30  (0.94-1.80) | 1.24  (0.91-1.70) | 1.20  (0.93-1.61) | 1.17  (0.90-1.65) | *0.005* |
| **High-Density Lipoprotein Cholesterol, mmol/L** | 1.30  (1.10-1.60) | 1.25  (1.03-1.50) | 1.30  (1.10-1.59) | 1.30  (1.10-1.60) | 1.37  (1.10-1.68) | 1.40  (1.19-1.71) | 1.40  (1.20-1.74) | *<0.001* |
| **Low-Density Lipoprotein Cholesterol, mmol/L** | 2.80  (2.12-3.50) | 2.90  (2.40-3.56) | 3.00  (2.31-3.64) | 2.81  (2.17-3.50) | 2.55  (1.90-3.26) | 2.42  (1.90-3.20) | 2.1  (1.90-3.23) | *<0.001* |
| **Non-High-Density Lipoprotein Cholesterol, mmol/L** | 3.36  (2.60-4.12) | 3.60  (2.90-4.26) | 3.60  (2.80-4.40) | 3.40  (2.60-4.20) | 3.09  (2.42-3.88) | 2.84  (2.03-3.60) | 2.90  (2.30-3.86) | *<0.001* |
| **Total cholesterol ≥5.0 mmol/L** | 3,311/7,367 | 666/1,387 | 919/1,760 | 801/1,707 | 552/1,458 | 296/854 | 77/201 | *<0.001* |
|  | (44.9%) | (48.0%) | (52.2%) | (46.9%) | (37.9%) | (34.7%) | (38.3%) |  |
| **Triglycerides ≥1.7 mmol/L** | 1,945/6,895 | 371/1,237 | 492/1,633 | 510/1,668 | 351/1,380 | 174/788 | 47/189 | *<0.001* |
|  | (28.2%) | (30.0%) | (30.1%) | (30.6%) | (25.4%) | (22.1%) | (24.9%) |  |
| **High-Density Lipoprotein Cholesterol <1.0 mmol/L for males or <1.3 mmol/L for females** | 2,311/8,927 | 529/1,719 | 584/2,150 | 524/2,102 | 414/1,728 | 212/997 | 48/231 | *<0.001* |
|  | (25.9%) | (30.8%) | (27.2%) | (24.9%) | (24.0%) | (21.3%) | (20.8%) |  |
| **Low-Density Lipoprotein Cholesterol, ≥4.9 mmol/L** | 183/7,023  (2.6%) | 31/1,241  (2.5%) | 62/1,643  (3.8%) | 56/1,694  (3.3%) | 19/1,439  (1.3%) | 12/828  (1.4%) | 3/178  (1.7%) | *<0.001* |

All values represent unadjusted, crude estimates.

# **Table 2.** Body mass index, blood pressure and lipid levels by area of residence

|  | **Total Subjects** | **Groups stratified by areas of residence** | | | | | | | ***p*** |
| --- | --- | --- | --- | --- | --- | --- | --- | --- | --- |
|  |  | **Bromley** | **Harrow** | **London NW** | **London SE** | **London SW** | **Southhall** | **Others** |  |
| **Body Mass Index, kg/m^2^** | 27.2  (24.2-31.1) | 27.9  (24.7-31.3) | 27.1  (24.1-30.8) | 27.2  (24.1-31.3) | 26.9  (24.0-31.5) | 27.5  (24.2-32.0) | 27.5  (24.6-31.5) | 27.8  (24.3-31.8) | *0.034* |
| **Body Mass Index groups** |  |  |  |  |  |  |  |  | *0.034* |
| **<18.5** | 166/10,093 | 5/320 | 106/5,891 | 24/2,156 | 9/258 | 7/597 | 10/566 | 5/305 |  |
|  | (1.6%) | (1.6%) | (1.8%) | (1.2%) | (3.5%) | (1.2%) | (1.8%) | (1.6%) |  |
| **18.5-25** | 2,966/10,093 | 80/320 | 1,745/5,891 | 651/2,156 | 80/258 | 173/597 | 147/566 | 90/305 |  |
|  | (29.4%) | (25.0%) | (29.6%) | (30.2%) | (31.0%) | (29.0%) | (26.0%) | (29.5%) |  |
| **25-30** | 3,783/10,093 | 117/320 | 2,264/5,891 | 803/2,156 | 84/258 | 203/597 | 218/566 | 94/305 |  |
|  | (37.5%) | (36.6%) | (38.4%) | (37.2%) | (32.6%) | (34.0%) | (38.5%) | (30.8%) |  |
| **≥30** | 3,178/10,093 | 118/320 | 1,776/5,891 | 678/2,156 | 85/258 | 214/597 | 191/566 | 116/305 |  |
|  | (31.5%) | (36.9%) | (30.1%) | (31.4%) | (32.9%) | (35.8%) | (33.7%) | (38.0%) |  |
| **Systolic Blood Pressure, mmHg** | 131  (120-142) | 131  (121-143) | 131  (120-142) | 132  (120-142) | 132  (120-144) | 132  (120-143) | 132  (120-144) | 130  (120-140) | *0.4* |
| **Diastolic Blood Pressure, mmHg** | 80  (71-85) | 78  (71-84) | 80  (71-85) | 79  (71-85) | 80  (72-86) | 80  (71-87) | 80  (72-85) | 79  (70-86) | *0.033* |
| **Blood pressure levels** |  |  |  |  |  |  |  |  | *<0.001* |
| **Systolic Blood Pressure <130 mmHg and Diastolic Blood Pressure <85 mmHg** | 2,509/16,518  (15.2%) | 44/340  (12.9%) | 1,610/10,028  (16.1%) | 351/2,814  (12.5%) | 50/375  (13.3%) | 174/1,169  (14.9%) | 212/1,319  (16.1%) | 68/476  (14.3%) |  |
| **Systolic Blood Pressure 130-140 mmHg and/or Diastolic Blood Pressure 85-90 mmHg, %** | 6,000/16,518  (36.3%) | 124/340  (36.5%) | 3,631/10,028  (36.2%) | 1,056/2,814  (37.5%) | 134/375  (35.7%) | 406/1169  (34.7%) | 461/1,319  (35.0%) | 188/476  (39.5%) |  |
| **Systolic Blood Pressure ≥140 and/or Diastolic Blood Pressure ≥90 mmHg** | 8,009/16,518  (48.5%) | 172/340  (50.6%) | 4,787/10,028  (47.7%) | 1,407/2,814  (50.0%) | 191/375  (50.9%) | 589/1169  (50.4%) | 646/1,319  (49.0%) | 220/476  (46.2%) |  |
| **Total cholesterol, mmol/L** | 4.80  (4.10.-5.60) | 5.00  (4.20-5.70) | 4.80  (4.00-5.53) | 4.84  (4.10-5.60) | 4.80  (4.10-5.43) | 4.90  (4.20-5.70) | 4.85  (4.03-5.61) | 5.00  (4.12-5.82) | *0.006* |
| **Triglycerides, mmol/L** | 1.27  (0.90-1.78) | 1.30  (0.90-1.70) | 1.28  (0.92-1.79) | 1.22  (0.90-1.72) | 1.10  (0.80-1.70) | 1.20  (0.90-1.70) | 1.35  (0.98-1.94) | 1.30  (0.80-1.74) | *0.002* |
| **High-Density Lipoprotein Cholesterol, mmol/L** | 1.30  (1.10-1.60) | 1.40  (1.10-1.80) | 1.30  (1.10-1.60) | 1.33  (1.10-1.60) | 1.47  (1.20-1.80) | 1.40  (1.10-1.69) | 1.21  (1.01-1.53) | 1.30  (1.12-1.56) | *<0.001* |
| **Low-Density Lipoprotein Cholesterol, mmol/L** | 2.80  (2.12-3.50) | 2.70  (2.00-3.40) | 2.80  (2.14-3.45) | 2.80  (2.11-3.55) | 2.80  (2.19-3.60) | 2.80  (2.03-3.50) | 2.80  (2.19-3.50) | 3.00  (2.24-3.61) | *0.3* |
| **Non-High-Density Lipoprotein Cholesterol, mmol/L** | 3.36  (2.60-4.12) | 3.20  (2.50-4.20) | 3.30  (2.55-4.10) | 3.47  (2.70-4.20) | 2.90  (2.40-3.90) | 3.29  (2.40-3.90) | 3.47  (2.71-4.29) | 3.55  (2.72-4.40) | *<0.001* |
| **Total cholesterol ≥5.0 mmol/L** | 3,311/7,367 | 109/213 | 1,919/4,372 | 575/1,257 | 63/152 | 251/523 | *285/636* | 109/214 | *<0.001* |
|  | (44.9%) | (51.2%) | (43.9%) | (45.7%) | (41.4%) | (48.0%) | (44.8%) | (50.9%) |  |
| **Triglycerides ≥1.7 mmol/L** | 1,945/6,895 | 47/162 | 1,146/4,031 | 319/1,187 | 33/116 | 136/537 | 215/688 | 49/174 | *<0.001* |
|  | (28.2%) | (29.0%) | (28.4%) | (26.9%) | (28.4%) | (25.3%) | (31.3%) | (28.2%) |  |
| **High-Density Lipoprotein Cholesterol <1.0 mmol/L for males or <1.3 mmol/L for females** | 2,311/8,927  (25.9%) | 49/204  (24.0%) | 1,362/5,363  (25.4%) | 389/1,569  (24.8%) | 31/188  (16.5%) | 106/545  (19.4%) | 303/806  (37.6%) | 71/252  (22.0%) | *<0.001* |
| **Low-Density Lipoprotein Cholesterol, ≥4.9 mmol/L** | 183/7,023  (2.6%) | 6/169  (3.6%) | 96/4,075  (2.4%) | 38/1,177  (3.2%) | 3/171  (1.8%) | 17/529  (3.2%) | 18/721  (2.5%) | 5/208  (2.4%) | *0.2* |

All values represent unadjusted, crude estimates.
